# Impact of GLP-1 Receptor Agonists in Gastrointestinal Endoscopy: An Updated Review

**DOI:** 10.3390/jcm13185627

**Published:** 2024-09-22

**Authors:** Sahib Singh, Saurabh Chandan, Dushyant Singh Dahiya, Ganesh Aswath, Daryl Ramai, Marcello Maida, Andrea Anderloni, Nicola Muscatiello, Antonio Facciorusso

**Affiliations:** 1 Internal Medicine, Sinai Hospital of Baltimore, Baltimore, MD 21215, USA; sahibs559@gmail.com; 2 Center for Interventional Endoscopy (CIE), Advent Health, Orlando, FL 32803, USA; saurabhchandan@gmail.com; 3 Gastroenterology & Hepatology, The University of Kansas School of Medicine, Kansas City, KS 66160, USA; 4 Gastroenterology & Hepatology, State University of New York Upstate Medical University, Syracuse, NY 13210, USA; aswathg@upstate.edu; 5 Brigham and Women’s Hospital, Harvard Medical School, Boston, MA 02115, USA; darylramai@gmail.com; 6 Department of Medicine and Surgery, School of Medicine and Surgery, University of Enna “Kore”, 94100 Enna, Italy; marcello.maida@unikore.it; 7 Gastroenterology and Digestive Endoscopy Unit, Fondazione IRCCS Policlinico San Matteo, 27100 Pavia, Italy; a.anderloni@smatteo.pv.it; 8 Gastroenterology Unit, Department of Medical and Surgical Sciences, University of Foggia, 71122 Foggia, Italy; antonio.facciorusso@virgilio.it; 9 Clinical Effectiveness Research Group, Faculty of Medicine, Institute of Health and Society, University of Oslo, 0372 Oslo, Norway

**Keywords:** glucagon-like peptide-1 receptor agonists, upper endoscopy, colonoscopy, capsule endoscopy

## Abstract

Glucagon-like peptide-1 receptor agonists (GLP-1 RAs) have become one of the most popular medications for patients with diabetes and obesity. Due to their effects on gut motility via central or parasympathetic pathways, there have been concerns about an increased incidence of retained gastric contents and risk of aspiration in the perioperative period. Hence, the American Society of Anesthesiologists (ASA) recommends holding GLP-1 RAs on the procedure day or a week before the elective procedure based on the respective daily or weekly formulations, regardless of the dose, indication (obesity or diabetes), or procedure type. On the contrary, the American Gastroenterological Association (AGA) advises an individualized approach, stating that more data are needed to decide if and when the GLP-1 RAs should be held prior to elective endoscopy. Several retrospective and prospective studies, along with meta-analyses, have been published since then evaluating the role of GLP-1 RAs in patients scheduled for endoscopic procedures. In this review, we discuss the current clinical guidelines and available studies regarding the effect of GLP-1 RAs on GI endoscopies.

## 1. Introduction

Glucagon-like peptide-1 receptor agonists (GLP-1 RAs) mimic the endogenous GLP-1 hormone in its insulin-promoting and glucagon-inhibiting actions [1]. The control of food-related glucose spikes along with a reduction in food intake due to inhibition of gastric emptying led to the approval of GLP-1 RAs for the treatment of diabetes and obesity [2,3]. The ease of use, great efficacy, and improvement in cardiovascular outcomes seen with GLP-1 RAs are reflected in their exponential use trend, with the growth rates reaching as high as 80% to more than 100% per month for commonly used formulations [4].

The effect of GLP-1 RAs on the digestive system has been an important area of recent discussion and research. The impact of GLP-1 RAs on lipid synthesis and oxidation in the liver, resulting in decreased inflammation and fibrosis, has led to studies evaluating their potential for the treatment of metabolic dysfunction-associated steatotic liver disease (MASLD), metabolic dysfunction-associated steatohepatitis (MASH), and inflammatory bowel disease (IBD), and the prevention of hepatobiliary and other gastrointestinal (GI) cancers [1,5]. On the other hand, GLP-1 RAs were found to alter the gastric/small bowel motility and appetite/satiation via central or parasympathetic (vagal-mediated) pathways, leading to an increased incidence of retained gastric contents (RGCs) with the associated aspiration risk during the perioperative period [6,7,8]. Substantial amounts of RGC were reported even after following the standard preoperative fasting period, as advised by the American Society of Anesthesiologists (ASA), or for longer durations in some cases [9,10]. Based on the available evidence at the time, the ASA released a statement advising holding GLP-1 RAs based on their dosing schedule in patients undergoing any type of elective procedures; however, this was challenged by the GI societies referring to the lack of data for conclusive opinions [6,11].

Several retrospective and prospective studies, along with meta-analyses, have been published since then describing various rates of RGCs and aspiration rates in patients on GLP-1 RAs, with some studies also advising on the appropriate time to hold these medications before elective procedures [12,13,14,15,16,17,18,19]. In this review, we discuss the current clinical guidelines and available studies regarding the effect of GLP-1 RAs on GI endoscopies.

## 2. Clinical Guidelines

The following consensus-based guidance was issued by the ASA Task Force on Preoperative Fasting in June 2023: (a) patients on GLP-1 RAs undergoing urgent/emergent procedures are to be considered ‘full stomach’; (b) hold GLP-1 RAs on the procedure day or a week before the elective procedure based on the respective daily or weekly formulations, regardless of the dose, indication (obesity or diabetes), or procedure type; (c) delay the elective procedure if the patient has symptoms such as nausea and vomiting; (d) ‘full stomach’ precautions to be taken if GLP-1RAs were not held as suggested before elective procedures [20]. 

Given the anticipated pushback from anesthesiologists in patients scheduled for GI endoscopies—along with insufficient and conflicting outcomes reported by the studies with respect to aspiration rates and the possibility of these effects being dependent on indication/dose of GLP-1 RAs—a multi-society statement was composed by the American Association for the Study of Liver Diseases (AASLD), American College of Gastroenterology (ACG), American Gastroenterological Association (AGA), American Society for Gastrointestinal Endoscopy (ASGE), and North American Society For Pediatric Gastroenterology, Hepatology, and Nutrition (NASPGHAN) [21]. The societies recommend that more data are needed to decide if and when the GLP-1 RAs should be held prior to elective endoscopy. Further, the impact of holding GLP-1 RAs is unknown, and the gastroenterologists are anyways familiar in managing symptomatic patients needing endoscopies. 

Subsequently, the AGA released a rapid clinical practice update advising an individualized approach prior to endoscopy, based on GLP-1RA indication, presence of symptoms, duration of fasting, and whether the endoscopy was required urgently [22]. If the patient was taking GLP-1 RA for weight loss, a single dose could be held, although it was uncertain whether gut motility would improve after holding just one dose. On the other hand, holding GLP-1 RA in diabetic patients could lead to poor glucose control, which could adversely affect the endoscopy outcomes [23]. In symptomatic patients with suspected RGCs, transabdominal ultrasound can be performed and rapid-sequence intubation could be considered in cases requiring urgent endoscopy [24]. Additionally, as combined upper and lower endoscopies have shown protective effects against RGCs, likely due to the liquid diet the previous day, the same dietary approach could be undertaken in patients on GLP-1 RAs instead of stopping the medication [25]. 

In addition to the GI societies, many anesthesiologists also expressed concern over the ASA’s recommendations. Ushakumari et al. suggested that there is currently no clear evidence regarding the duration for which GLP-1 RAs must be held or the adequate fasting time in this patient population [26]. Milder et al. agreed with the AGA regarding the risks of holding GLP-1 RAs in diabetic patients, while also suggesting a holding period of at least three half-lives before any elective procedure in obese patients [27].

## 3. Clinical Studies

### 3.1. Upper Endoscopy

**Figure 1:**
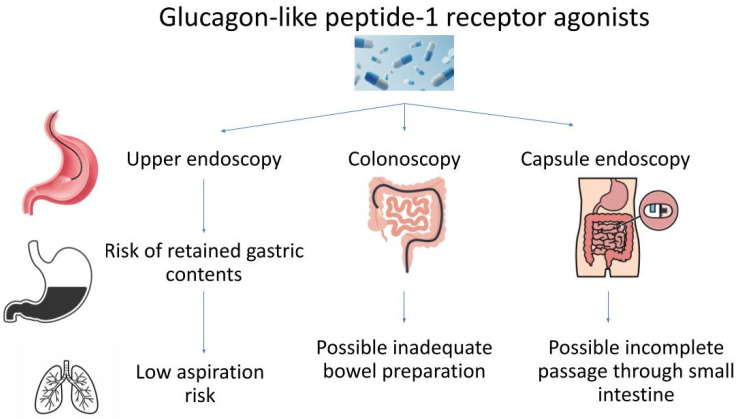
Impact of GLP-1 RAs on endoscopy.

Stark et al. conducted a retrospective cohort study of patients on GLP-1 RAs (*n* = 59) undergoing esophagogastroduodenoscopy (EGD) and compared them with matched controls (*n* = 118) (Figure 1 and Table 1) [28]. Retained food content was found in four patients in the GLP-1 RA group vs. two in the control group, without any significant difference between the groups (6.8% vs. 1.7%, *p* = 0.08). One patient in each group required lavage (1.7% vs. 0.8%, *p* = 0.62), and none of the patients needed repeat EGD. The study was limited due to low sample size, possible missed recording of retained food, variable dose, treatment duration, and adherence to GLP-1 RAs. 

In a single-arm retrospective analysis by Firkins et al., clinical outcomes in GLP-1 RA users undergoing EGD were assessed [29]. Of the 1512 EGDs included in the study, 142 were found to have RGCs (9.4%), with solid retained contents being present in 112 cases (7.4%) and liquid contents in 27 cases (1.6%). Around 93 patients (11.6%) had upper GI symptoms such as nausea, vomiting, and abdominal pain. The procedure was aborted in 30 cases (2%), with 21 cases requiring a repeat endoscopy (1.4%). Four patients had intraprocedural hypoxia (0.3%), two experienced aspiration (0.1%) and three (0.2%) had other adverse events such as bronchospasm and cholangitis. Univariate analysis revealed that younger age was associated with an increased RGC risk (*p* = 0.003), whereas colonoscopy performed on the same day exerted a protective effect against RGC (*p* < 0.001). Multivariate analysis confirmed the roles of young age (*p* = 0.026) and same-day colonoscopy (*p* < 0.001). While some GLP-1 RAs, including tirzepatide and oral semaglutide, demonstrated a higher RGC risk in univariate analysis, this was not reflected in the subsequent multivariate results. Timing of the procedure or anesthesia type did not have any individual effect over the RGCs. Procedure discontinuation and requirement of repeat endoscopy were significantly associated with the presence of RGCs (*p* < 0.001 for both). 

In an observational study from the Mayo Clinic Health System, Anazco et al. found only two cases of pulmonary aspiration among 4134 endoscopies in patients on GLP-1 RAs, amounting to a rate of 4.8 per 10,000 endoscopies [30]. This rate was similar to the previous study from the same hospital system conducted in a period before GLP-1 RAs became popular (2010–2016), with 4.6 cases of aspiration per 10,000 endoscopies. In another case series by Maselli et al., 57 patients on GLP-1 RAs scheduled for endoscopic sleeve gastroplasty were instructed to maintain a liquid-only diet for 24 h and a fasting period of 12 h prior to the procedure [31]. No cases of retained gastric solids, pulmonary aspiration, regurgitation of gastric contents, or hypoxia were observed.

Silveira et al. performed a retrospective analysis of patients having elective upper endoscopy and stratified the cases based on semaglutide use within 30 days before the procedure [25]. Of the 404 patients—with 33 in the semaglutide group and 371 in the non-semaglutide group—increased RGC was higher in the former group (24.2% vs. 5.1%, *p* < 0.001). In the unadjusted analysis, increased RGC was found to be associated with semaglutide use (*p* < 0.001) and ongoing GI symptoms (*p* < 0.001). On the contrary, RGCs were lower in patients who had combined upper endoscopy and colonoscopy (*p* = 0.011). Patients with or without increased RGCs had no difference pertaining to the fasting duration for clear liquids (*p* = 0.084) or solids (*p* = 0.457). Subsequent propensity weighted analysis also showed similar effects of semaglutide use, GI symptoms, and combined procedures on RGCs (*p* < 0.001 for all). Interestingly, the amount of RGC found did not correlate with semaglutide use (*p* = 0.99). Further, only one aspiration event was noted; the patient was on semaglutide prior to the endoscopy but did not have any ongoing GI symptoms. 

In a larger sample size study by Nadeem et al., conducted at Geisinger Medical Center (2019–2023), a total of 35,183 patients undergoing upper endoscopy were divided into GLP-1 (*n* = 922) and non-GLP-1 groups (*n* = 34,261) [32]. The former group had older patients (57.1 ± 12.9 vs. 53.9 ± 17.5 years, *p* < 0.0001), with a higher average body mass index (BMI) (36.4 ± 8.9 vs. 30.5 ± 7.8 kg/m^2^, *p* < 0.0001) and proportion of diabetic patients (756 vs. 5407, *p* < 0.0001). As compared to the patients not on GLP-1 RAs, those using these medications had higher RGC rates (13.6% vs. 2.3%, *p* < 0.0001), aborted procedures (1.5% vs. 0.3%, *p* < 0.0001), and requirements for repeat EGD (2.4% vs. 1.1%, *p* = 0.0001). Multiple logistic regression performed after adjustments for demographics and comorbidities confirmed the higher risk of RGCs (4 times), aborted procedures (4 times), and need for repeat EGD (2 times) among patients on GLP-1 RAs. Upon stratifying the patients based on presence of diabetes, GLP-1 RAs were associated with increased RGCs and aborted procedures in both diabetic and non-diabetic patients. While repeat EGDs were higher among GLP-1 RA users who were diabetic, there was no significant difference compared to the non-GLP-1 RA group in patients without diabetes (*p* = 0.655). Postprocedural adverse events such as bronchospasm did not differ between the GLP-1 RA users and non-users (0.2% vs. 0.2%, *p* = 0.707). The one aspiration event reported was in the non-GLP-1 RA cohort. 

In the 1:1 matched case-control study by Chapman et al., patients scheduled for EGD and on GLP-1 RAs were compared with controls (not on GLP-1 RAs) [33]. Among the 84 patient pairs included, the GLP-1 RA group had higher BMI (40.70 ± 13.29 vs. 31.23 ± 10.65 kg/m^2^, *p* < 0.001), no underweight patients (0 vs. 7.1%, *p* < 0.001), and a greater number of patients who received monitored anesthesia care (MAC) (76.2% vs. 58.3%, *p* = 0.014). Clean gastric mucosa was less common (57.1% vs. 67.9%) along with higher RGCs (13.1% vs. 4.8%, *p* = 0.025) in the GLP-1 RA users. Similarly, the gastric mucosal visibility score (POLPREP) was lesser in patients on GLP-1 RAs compared to the control group (2.14 ± 1.03 vs. 2.57 ± 0.74, *p* = 0.0012). Further, BMI increase by every unit led to a lower visibility score (*p* < 0.001). Logistic regression revealed the association of GLP-1RA use with lower mucosal visibility score (*p* < 0.001), without any significant difference among the various GLP-1 RA medications. While the GLP-1 RA group had a higher number of aborted EGDs (4.8% vs. 0, *p* = 0.043), no differences were observed compared to the non-GLP-1 RA group with respect to adverse events (procedure- or anesthesia-related) or mortality at 30 days.

Barlowe et al. conducted a retrospective cohort study based on the nationwide claims database of diabetic patients who had an upper endoscopy during the period of 2005–2021 [34]. The outcomes were compared between GLP-1 RA (*n* = 15,119), dipeptidyl peptidase 4 inhibitor (DPP4i, *n* = 14,407), and chronic opioid (*n* = 7257) users. The opioid group had a higher comorbidity burden, including asthma, chronic obstructive pulmonary disease (COPD), and 2+ Charlson Comorbidity Index. The three groups had comparable rates of aspiration (0.05% vs. 0.07% vs. 0.11%) and aspiration pneumonia (0.07% vs. 0.07% vs. 0.11%). The GLP1-RA group had a lower risk of pneumonia (0.18% vs. 0.59%), respiratory failure (0.10% vs. 0.41%), and composite pulmonary adverse events (0.31% vs. 1.01%) when compared with the chronic opioid group. Further, post-EGD hospitalization and emergency room visits were both lower in patients on GLP-1 RAs vs. DPP4is/opioids (2.22% vs. 2.33%/4.93% and 4.15% vs. 4.94%/10.69%, respectively). Sensitivity analyses accounting for baseline risk factors and stricter use criteria showed consistent results.

In the multicenter cross-sectional study by Phan et al., patients (*n* = 815) who underwent upper endoscopy while being on GLP-1 RAs were analyzed [35]. RGCs were present in 8.6% of cases, with the amount being minimal in 14.3%, moderate in 44.3%, and large in 41.4% of patients. Unplanned intubation was required in only one patient due to a large RGC, and the procedure was aborted in fourteen patients. As compared to those without RGCs, patients with RGCs had higher odds of being diabetic (*p* = 0.009) and on insulin therapy (*p* < 0.01), with lesser likelihood of holding GLP-1 RAs (*p* < 0.001). Among diabetic patients, male gender (*p* = 0.01) and absence of gastroparesis (*p* = 0.004) were associated with RGCs. Furthermore, those with uncontrolled diabetes had higher chances of having RGCs compared with other diabetic groups (*p* = 0.006). When grouped based on the ASA recommendations, patients who held their GLP-1 RAs had lesser RGCs compared to those who did not hold (4.4% vs. 12.7%, *p* < 0.001); however, holding the GLP-1 RAs did not affect the quantity retained (*p* = 0.37), intubation rate (*p* = 0.53), or procedure abortion rate (*p* = 0.40). Multivariate analysis showed an association of RGCs with not holding GLP-1 RAs (*p* < 0.001), an HbA1C increase by every 1% (*p* < 0.001), and preoperative glucose levels >150 mg/dL (*p* = 0.002). 

Garza et al. conducted a case-control study where patients on GLP-1 RAs undergoing upper endoscopy had a higher rate of gastric residue as compared with controls (14% vs. 4%, *p* < 0.01) [36]. This was further shown in the subgroups of diabetic patients (14% vs. 4%, *p* < 0.01), those on insulin (17% vs. 5%, *p* < 0.01), and those with diabetic complications (15% vs. 2%, *p* < 0.01). Prolonged fasting and maintenance of a clear liquid diet for colonoscopy (*p* < 0.01) and performing the procedure in the afternoon (*p* < 0.01) lowered the risk of gastric residue. No aspiration events occurred in any of the procedures. 

In another cohort study by Wu et al., gastric contents were observed in 18.7% of EGDs in patients on GLP-1 RAs vs. 4.9% in the control group (*p*  =  0.004) [37]. The GLP-1 RA group had more procedures with endotracheal anesthesia (21% vs. 11%), with an additional four cases being converted from MAC due to presence of RGCs (6% vs. 0). One aspiration event was recorded in the GLP-1 RA group vs. none in the control group. Differentiating the GLP-1 RA users into groups of patients on regular diet vs. clear liquid/low-residue diet for 24 h prior to the endoscopy, with fasting from midnight, Ghazanfar et al. reported a higher residual food rate among those on regular diet (10% vs. 1.5%, *p* = 0.03) [38]. Further, four patients who had residual food and ongoing GI symptoms were on a regular diet. No aspiration events were reported.

### 3.2. Colonoscopy

In an observational study by Tong et al., patients with diabetes undergoing colonoscopy (bowel preparation with polyethylene glycol) were divided into liraglutide (GLP-1 RA), sitagliptin (DPP-4i), and control groups [39]. Inadequate bowel cleaning was not statistically different across the three groups (17.5% vs. 20.5% vs. 21.7%, *p* = 0.927); similarly, the mean Boston Bowel Preparation Scale (BBPS), cecal intubation time, and rates of polyp detection were comparable (*p* > 0.0.05). However, liraglutide use was associated with increased GI symptoms (such as nausea, vomiting; *p* < 0.05) and a higher incidence of inadequate bowel cleaning in a subgroup of patients with diabetic peripheral neuropathy (61.3% vs. 32.1% sitagliptin, *p* = 0.022; 61.3% vs. 32.8% control, *p* = 0.025). Along the same lines, Yao et al. reported patients in the GLP-1 RA group to have a lower mean BBPS (*p* = 0.046), along with a higher proportion of patients with BBPS < 5 (*p* = 0.01) and those requiring repeat colonoscopy due to inadequate bowel preparation (*p* = 0.041) [40].

### 3.3. Capsule Endoscopy

Nakatani et al. assessed GI motility and residue rates via capsule endoscopy in 14 diabetic patients before and after administration of liraglutide [41]. While the gastric transit time in patients with diabetic neuropathy (DN) was not impacted significantly due to liraglutide (*p* = 0.19), it was significantly increased after liraglutide administration in the non-DN group (*p* = 0.03). A similar trend was noticed in the duodenal and small intestine transit time as well. GI residue rate was higher in both DN and non-DN groups after receiving liraglutide (*p* < 0.001). In the recent matched cohort study of patients with diabetes undergoing capsule endoscopy by Odah et al., the GLP-1 RA group had increased gastric transit time (99.3 ± 134.2 min vs. 25.3 ± 31.6 min, *p* < 0.001) and a greater rate of incomplete passage through the small intestine (23.5% vs. 4.4%, *p* < 0.01) as compared with the control group [42].

### 3.4. Meta-Analyses

Hiramoto et al. conducted a meta-analysis to evaluate the effect of GLP-1 RAs on gastric emptying [8]. Among the studies quantifying gastric emptying by scintigraphy, GLP-1 RA use caused longer t1/2 compared to placebo (138.4 vs. 95.0 min, *p* < 0.01). While the type of GLP-1 RA formulation or duration of treatment did not affect gastric emptying (scintigraphy), it was significantly increased in obese patients with BMI ≥ 30 kg/m^2^ (*p* = 0.018). No significant difference was noted between GLP-1 RA and placebo groups with respect to gastric emptying measured via the acetaminophen absorption test (*p* = 0.432). The study concluded that a gastric emptying delay of around 36 min (scintigraphy) in patients on GLP-1 RAs would not have a great impact on the standard fasting time of 6–8 h, and hence these medications can be continued in the perioperative period. Further, a liquid diet does not delay gastric emptying (as seen with the acetaminophen absorption test) and can be given on the day prior to the endoscopy. 

In the meta-analysis by Facciorusso et al., 13 studies (84,065 patients) were included demonstrating the effects of GLP-1 RAs on upper endoscopy [43]. As compared to non-users, the GLP-1 RA users had significantly higher RGC rates (odds ratio [OR] 5.56, 95% CI 3.35–9.23), and the results were consistent in the subgroup analysis of full-text papers—those with propensity score matching, diabetic patients, and fasting duration (greater or less than 12 h). While the GLP-1 RA group had a higher rate of aborted procedures (1% vs. 0.3%) and need for repeat procedures (2% vs. 1%), no significant differences were noted with regards to adverse events (0.3% vs. 0.1%) or bronchial aspiration (0.3% vs. 0.2%).

## 4. Controversy

Several controversies have emerged regarding the impact of GLP-1 RAs on endoscopy outcomes [44,45,46]. First, the gastric emptying methodologies in pharmacokinetic studies have been variable [8]. Although the acetaminophen absorption test has been widely used for surrogate evaluation of liquid-phase emptying, it is not as reliable as scintigraphy, which is the gold standard diagnostic test [12]. Further, the retention rate at 4 h was reported to be 7% before and 37% after initiation of GLP-1 RAs in one of the two randomized studies reporting the outcome at this time frame [13,14]. Second, there is a difference between the effects of GLP-1 RAs according to the oral or injectable administration method. The side effects of injectable semaglutide are lower than those of oral administration and than other older classes of GLP-1 RAs. Third, the short-acting GLP-1RAs have more effect on the gastric motility compared to long-acting formulations. Fourth, the doses are also important because for diabetics the dose is lower than for obesity, and implicitly, the adverse effects are lower.

## 5. Limitations and Future Perspectives

The currently available evidence is limited by the underlying heterogeneity in patient characteristics, comorbidities, study designs, and practice protocols of the participating institutions. Given the confusion regarding the best practice for patients undergoing endoscopic procedures and on GLP-1 RAs, and given the increased prescription of these drugs for several indications [47], a rapid increase in the number of clinical studies has occurred. Results from ongoing and future studies would likely help in clarifying the perioperative management of these patients, particularly in light of the recent advances and the new procedures introduced in gastrointestinal endoscopy [48,49,50,51,52].

## 6. Conclusions

GLP-1 RAs seem to adversely affect gut motility, with an increased risk of RGCs during upper endoscopy (without a simultaneous increase in aspiration risk), inadequate bowel preparation during colonoscopy, and incomplete passage through the small intestine during capsule endoscopy. The findings from these studies support the tailored approach to every GLP-1 RA user scheduled for endoscopy, as mentioned by GI societies and anesthesiologists apart from the ASA. 

## Figures and Tables

**Table 1:** Major clinical studies evaluating GLP-1 RA effects on upper endoscopy.

Reference	Age (y)	Male %	Race/Ethnicity %	BMI (kg/m^2^)	DM %	GLP-1 RA Dose	GLP-1 RA Route	Findings
[25]	50.8	51.5	-	26.2	9.4	-	Subcutaneous	RGCs in the GLP-1 RA vs. control group (24.2% vs. 5.1%, *p* < 0.001), only 1 aspiration event in the GLP-1 RA group
[28]	65	88.5	-	33	97.5	-	Subcutaneous	RGCs in the GLP-1 RA vs. control group (6.8% vs. 1.7%, *p* = 0.08)
[29]	60.9	35.8	-	35.2	76.7	-	Subcutaneous or oral	RGCs (9.4%), aspiration (0.1%)
[30]	-	-	-	-	-	-	-	4.8 aspiration cases per 10,000 endoscopies
[31]	44	10.5	-	40.1	35.1	Semaglutide 0.25–2.4 mg/week Liraglutide 0.6–3 mg/day Dulaglutide 0.75–4.5 mg/week Tirzepatide 2.5–15 mg/week	Subcutaneous	No cases of RGCs or pulmonary aspiration
[32]	54	41	White 91 Hispanic 5 Black 2	30.7	18	-	-	RGCs in the GLP-1 RA vs. control group (13.6% vs. 2.3%, *p* < 0.0001), only 1 aspiration event in the control group
[33]	53.94	29.8	White 60.1 Black 39.9	35.96	85.7	-	-	RGCs in the GLP-1 RA vs. control group (13.1% vs. 4.8%, *p* = 0.025)
[34]	56	45	-	-	100	-	-	Aspiration in the GLP-1 RA, dipeptidyl peptidase 4 inhibitor, and chronic opioid users (0.05% vs. 0.07% vs. 0.11%)
[35]	60.7	42.3	Caucasian 53.9 African American 19.6 Hispanic 17.5 Asian 3.1	-	82.5	-	Subcutaneous or oral	RGCs (8.6%)
[36]	61.5	50.5	-	32.45	88	-	Subcutaneous or oral	RGCs in the GLP-1 RA vs. control group (14% vs. 4%, *p* < 0.01), no aspiration events
[37]	61.3	42.5	-	34	47	-	-	RGCs in the GLP-1 RA vs. control group (18.7% vs. 4.9%, *p* = 0.004), 1 aspiration event in the GLP-1 RA group vs. 0 in the control group
[38]	60	63.1	-	-	85.6	-	-	RGCs in the regular diet vs. clear liquid/low-residue diet groups (10% vs. 1.5%, *p* = 0.03), no aspiration events

BMI—body mass index, y—years, kg/m^2^—kilograms divided by height in meters squared, DM—diabetes mellitus, RGCs—retained gastric contents.

## Data Availability

The data presented in the study are openly available in online databases.
